# Virtual screening for oseltamivir-resistant a (H5N1) influenza neuraminidase from traditional Chinese medicine database: a combined molecular docking with molecular dynamics approach

**DOI:** 10.1186/2193-1801-2-115

**Published:** 2013-03-18

**Authors:** Vasudevan Karthick, Karuppasamy Ramanathan

**Affiliations:** Bioinformatics Division, School of Bio Sciences and Technology, Vellore Institute of Technology, Vellore, Tamil Nadu 632014 India

**Keywords:** Oseltamivir-resistance, Traditional Chinese medicine database (TCMD), Virtual screening, Molecular docking, Molecular dynamic simulation

## Abstract

The neuraminidase (NA) of the influenza virus is the target of antiviral drug, oseltamivir. Recently, cases are reported that Influenza virus becoming resistant to oseltamivir, necessitating the development of new long-acting antiviral compounds. Most importantly, H274Y mutation in neuraminidase exhibits high levels of resistance to oseltamivir. In this report, a novel class of lead molecule with potential NA inhibitory activity was found from the traditional Chinese medicine database (TCMD) using virtual screening approach. Initially ADME properties of the lead compounds were analyzed with respect to the Lipinski rule of five. Subsequently, the data reduction was carried out by employing molecular docking study. Final validation was done by means of molecular dynamic simulations. The toxicity profiles for the screened compound were also analyzed. The result indicates that neoglucobrassicin (a compound derived from TCMD) become a promising lead compound and be effective in treating oseltamivir-resistant influenza virus strains.

## Introduction

Avian influenza A (H5N1) virus is highly pathogenic which leads to high mortality rate in humans. Influenza A viruses are enveloped RNA viruses with an eight-segmented, single-stranded, belonging to the family Orthomyxoviridae (Malik Peiris et al. 2009). Influenza viral membrane composed of two main glycoproteins the hemagglutinin (HA) and neuraminidase (NA) (Zhou et al. 2009). Based on antigenic properties influenza type A viruses were classified into 16 HA (H1–H16) and 9 NA (N1–N9) subtypes (Gamblin & Skehel 2010). In humans influenza viruses binds to cell sialic acid linked to galactose by an α-2,6 linkage (SA α-2,6) (Malik Peiris et al. 2009). Hemagglutinin (HA) is the receptor-binding and membrane fusion glycoprotein of influenza virus and it contains two subunits which have different functions. HA_2_ mediates fusion of membrane and viral entry, while HA_1_ is involved in binding to the sialic acid receptors on the target cell (Skehel & Wiley 2000), and neuraminidase has sialidase activity in which it cleaves sialic acid residues and facilitates release of virus progeny by removing the terminal sialic acids to which the HA binds (Stephenson & Democratis 2006).

Neuraminidase (NA) inhibition is a pivotal step in restricting the spread of influenza virus infection in the host. Oseltamivir (Tamiflu) and zanamivir (Relenza) are two currently used NA inhibitors that were developed using the knowledge of the enzyme structure. It has been proposed that closer the inhibitors resemble the natural substrate, the less likely they are to select drug-resistant mutant viruses that retain viability. Oseltamivir is the first line antiviral drug used for the treatment against influenza and it is an active influenza neuraminidase inhibitor which is recommended for treating and preventing influenza virus infection. However, there have been reports of oseltamivir-resistant mutant selection in vitro and from infected humans. In particular H274Y, the principal mutation isolated in association with oseltamivir treatment that is specific to the N1 group (Yen et al. 2007) and that has recently been shown to be present in substantial numbers of H5N1 viruses isolated from humans (Uyeki 2009). Virtual screening (VS) is a widely used method that has been shown to be successful in a variety of studies (Oprea & Matter 2004). In the past few years, many reports indicated that virtual screening techniques proved to be effective in making qualitative predictions that discriminated active from inactive compounds (Chen 2008; Chang et al. 2011a; Chang et al. 2011b; Lin et al. 2011; Chang et al. 2011c; Chang et al. 2011d). The basic goal of the virtual screening is the reduction of the enormous virtual chemical space of small organic molecules, to synthesize and/or screen against a specific target protein, to a manageable number of the compound that inhibit a highest chance to lead to a drug candidate.

The use of experimentally derived protein structures and a hybrid computational method that combines the advantages of docking algorithms with dynamic structural information provided by molecular dynamics simulation techniques has been successfully applied to a number of systems and most recently, aid in the discovery of HER2, H1N1 and M2 inhibitors (Yang et al. 2011; Tsai et al. 2011; Lin et al. 2011a; Lai et al. 2011; Chang et al. 2011e; Lin et al. 2011b). In addition, molecular dynamic simulation could also be helpful in the analysis of mechanism of drug resistance. Recently, we have identified the mechanism of oseltamivir resistance by means molecular docking and molecular dynamics approach (Karthick et al. 2012). The available literature evidences suggests that two or three Arginine residues (150 loop) surrounding the carboxylic group of NA inhibitors play a key role in orienting and stabilizing various inhibitors (Russell et al. 2006). Our computational approach also correlates well with the experimental evidences. It indicates that the existence of intermolecular interactions in some of the key Arginine residues present in the cavity of NA play a key role in stabilizing the inhibitors (Karthick et al. 2012).

Hence, the goal of the present study is to screen a virtual library of oseltamivir analogs, which fill also the cavity adjacent to the active site, aiming at finding new potent NA inhibitors of the avian influenza virus H5N1 with the aid of Traditional Chinese medicine database (TCMD). Therefore, a combined molecular docking and molecular simulation approach has been applied to screen the potential molecule from TCM. TCM has been in use in China for thousands of years and is still very prominent in medical field today. TCM pays more emphasis on systemic medication and subscribes medicines originated from natural herbs that give little or none side effects. Various research have been done on studying Chinese herbal medicine and studies suggest that it can aid in the cancer treatments especially head and neck cancer (Tou & 2012Chen ), solution for the stroke risk (Chang et al. 2011f; Chen et al. 2011; Chen & Chen 2011) and also for the flu treatment (Chang et al. 2011c; Chang et al. 2011d; Lin et al. 2011a; Lai et al. 2011; Chang et al. 2011e; Lin et al. 2011b; Chang et al. 2011g). However, none of the earlier study reported the lead compound for the drug resistant target of influenza virus (H5N1).

With the emergence of oseltamivir-resistant influenza virus infections, compounds with alternative binding modes may be of great clinical implications. Hence, in this work, a combined molecular docking and dynamics approach has been applied to screen the potential molecule from TCM against the drug resistant target of NA by employing oseltamivir as query. Hopefully, we have proposed some useful candidates for H5N1 and put forward a constructive concept of designing H5N1 inhibitors.

## Materials and methods

### Data set preparation

The native and mutant (H274Y) type coordinates of NA were taken from the Brookhaven Protein Data Bank (Berman et al. 2000). The corresponding PDB codes were 2HU4 and 3CL0 respectively (Russell et al. 2006). The structures were solved with 2.50 and 2.20 Å resolutions, respectively, having residues from 83 to 468. Oseltamivir was used as the small molecule/inhibitor for our investigation. The SMILES strings were collected from PubChem, a database maintained in NCBI (Feldman et al. 2006), and submitted to CORINA for constructing the 3D structure of the small molecule (Gasteiger et al. 1990). The three dimensional structure of target proteins (2HU4 and 3CL0) and drug molecule were energy-minimized using GROMACS package 4.5.3 (Hess et al. 2008; Spoel et al. 2005) adopting the GROMOS43a1 force field parameters before performing the computational analysis.

### Virtual screening

Virtual screening (Shoichet 2004) is the computational analogue of biological screening. The approach has become increasingly popular in the pharmaceutical research for lead identification. The basic goal of the virtual screening is the reduction of the massive virtual chemical space of small organic molecules, to screen against a specific target protein, to a manageable number of the compound that inhibit a highest chance to lead to a drug candidate (Tondi et al. 1999). We obtained 3D structure of oseltamivir in SDF format from PubChem database and we submitted the structure in TCM database choosing similarity option in order to get most similar structure of oseltamivir (Chen 2011).

### ADME

Molecular properties such as membrane permeability and bioavailability are always associated with some basic molecular descriptors such as logP (partition coefficient), molecular weight (MW), or counts of hydrogen bond acceptors and donors in a molecule (Ertl et al. 2000). These molecular properties were used in formulating “rule of five” (Lipinski et al. 1997) The rule states that most molecules with good membrane permeability have Molecular Weight ≤ 500, calculated octanol–water partition coefficient, log P ≤ 5, hydrogen bond donors ≤ 5 and acceptors ≤ 10 (Muegge 2003). Therefore, Lipinski’s Rule of Five was used to test the bioavailability characteristics such as absorption, distribution, metabolism, elimination (ADME) of the lead compounds. In the present study, these molecular properties for all the lead compounds were estimated by using MOLINSPIRATION program (2008).

### Computation of docking energy

The lead compounds obtained from the virtual screening analysis were used in the docking calculation. Flexible docking simulation was performed using ArgusLab 4.0.1 (Thompson 2004). The ArgusLab 4.0.1 docking program has been extensively validated with docking accuracy at ~3 Å, for root mean square deviation (RMSD) value between the predicted and original crystallographic pose. Flexible ligand docking of ArgusLab is available by describing the ligand as a torsion tree. Groups of bonded atoms that do not have rotatable bonds are nodes, while torsions are the connections between the nodes. Topology of a torsion tree is a determinative factor influencing efficient docking. In the docking calculations, the scoring method Ascore from the ArgusLab 4.0.1 suite is employed. AScore is based on the decomposition of the total protein–ligand binding free energy, taking into account the following contributions: the van der Waals interaction between the ligand and the protein, the hydrophobic effect, the hydrogen bonding between the ligand and the protein, the hydrogen bonding involving charged donor and/or acceptor groups, the deformation effect, and the effects of the translational, and rotational entropy loss in the binding process, respectively, (Thompson 2004). The AScore function, with the parameters read from the AScore.prm file, was used to calculate the binding energies of the resulting docked structures. This file contains the coefficients for each term in the scoring function. Each docking run was repeated five times to get the best results. A maximum of 150 poses were allowed to be analyzed. Given the imperfections of computational docking analysis, a recent trend in this field has been the introduction of re-dockings to evaluate the ligand binding affinity. The results from these docking studiess were then combined to balance the errors in the *in silico* prediction and this kind of approach certainly improve the probability of identifying ‘true’ ligands and helpful in the ligand binding analysis. For all docking analysis, the binding site residues were retrieved from the program called ligand Contact Tool (LCT) (Lopez et al. 2007) by employing the NA-Oseltamivir complex structure as query. Moreover, literature evidences were also used to screen and validate the active site residues (Colman et al. 1983). LIGPLOT (Wallace et al. 1995) used to visualize the interactions exists in the complex structures.

### Molecular dynamics simulation

The crystal structure of wild NA-oseltamivir complex (2HU4), mutant NA-oseltamivir complex (3CL0) and docked complex of mutant-NA neoglucobrassicin complex were used as starting point for MD simulation using GROMACS package 4.5.3 (Hess et al. 2008; Spoel et al. 2005) adopting the GROMOS43a1 force field parameters. The structures were solvated in cubic 0.9 nm, using periodic boundary conditions and the SPC water model (Meagher & Carlson 2005). PRODRG server (Schuttelkopf & Van Aalten 2004) was used to generate ligand topology. 3Na + counter ions were added to neutralize the total charge of the system. 1000 steps of steepest descent energy minimization were carried out for the protein-ligand complex. After energy minimization, the system was equilibrated at 300 k. The equilibrated structures were then subjected to molecular dynamic simulations for 5000 ps at the constant temperature of 300 K and at the constant pressure of 1 atm, and the integration time step was set to 2 fs. The non-bonded list was generated, using an atom-based cutoff of 8 Å. The long range electrostatic interactions were handled by the particle-mesh Ewald algorithm (Darden et al. 1999). 0.9 nm cut-off was employed to Lennard-Jones interaction. During the simulations, all bond lengths containing hydrogen atoms were constrained utilizing the Lincs algorithm (Lindahl et al. 2001), the trajectory snapshots were stored for structural analysis at every pico-second. Root mean square deviation (RMSD) was analyzed through Gromacs utilities g_rmsd.

### Toxicity

Successful drug discovery requires high quality lead structures which may need to be more drug-like than commonly accepted (Proudfoot 2002). Toxicity and poor pharmacokinetics should be eliminated in the early stages of drug discovery. Hence, the hits were further screened using drug-likeliness, drug score and toxicity characteristics. These physico-chemical properties were therefore calculated for the filtered set of hits using the programs (OSIRIS 2001).

The OSIRIS program calculates the drug likeliness based on a list of about 5,300 distinct substructure fragments created by 3,300 traded drugs as well as 15,000 commercially available chemicals yielding a complete list of all available fragments with associated drug likeliness. The drug score combines drug-likeliness, cLogP, logS, molecular weight, and toxicity risks as a total value which may be used to judge the compound’s overall potential to qualify for a drug.

## Results and discussion

### Virtual screening and bioactivity analysis

The new lead molecules were screened from using currently active oseltamivir compound as template from the TCMD and 9 compounds were founded. Pharmacokinetic & toxicity issues are blamed for more than half of the failure in the clinical trials. Therefore in the first part of the virtual screening evaluates the drug likeness of the same molecules most independent of their intended drug target. The molecular properties and bioactivity for the lead compounds were predicted using Molinspiration program (http://www.molinspiration.com). The LogKow program (Remko 2009) estimates the log octanol/water partition coefficient (logP) of organic chemicals and drugs by an atom/fragment contribution method developed at Syracuse Research Corporation (Wang et al. 1997). Log P value is an important predictor of per oral bioavailability of drug molecules (Clark 1999; Chang et al. 2004). Therefore, we calculated log P values along with other physiochemical properties such as molecular mass, the number of hydrogen bond acceptors and the number of hydrogen bond donors for the all the 9 lead compounds obtained from the TCM database. The results showed that 4 molecules have zero violations of the Rule of 5 which suggest that these molecules likely to have good bioavailability (Table 1). Hence, the subsequent analysis was performed with the help of these screened molecules.

**Table 1:** **Calculations of molecular properties of oseltamivir and lead compound using Molinspiration**

S. No	Compounds	miLogP	MW	nON	nOHNH	nrotb	nviolations
1	Oseltamivir	0.852	315.38	6	3	8	0
**2**	**Neoglucobrassicin**	**-3.619**	**477.493**	**10**	**3**	**8**	**0**
**3**	**Acanthoglabrolide**	**3.502**	**418.486**	**7**	**0**	**7**	**0**
4	Epifriedelanol Acetate	8.556	470.782	2	0	2	1
5	g-strophanthin	-2.178	584.659	12	8	4	3
**6**	**Allyl Propyl disulfide**	**2.86**	**148.296**	**0**	**0**	**5**	**0**
**7**	**Allyl monosulfide**	**2.127**	**114.213**	**0**	**0**	**4**	**0**
8	Strictinin	0.312	634.455	18	11	3	3
9	Strophanthin	-1.421	726.813	15	7	8	3
10	Acanthoside	-1.475	742.724	18	8	12	3

Bold indicates ADME screened compounds based on Lipinski rule of 5.

### Molecular docking studies

**Figure 1:**
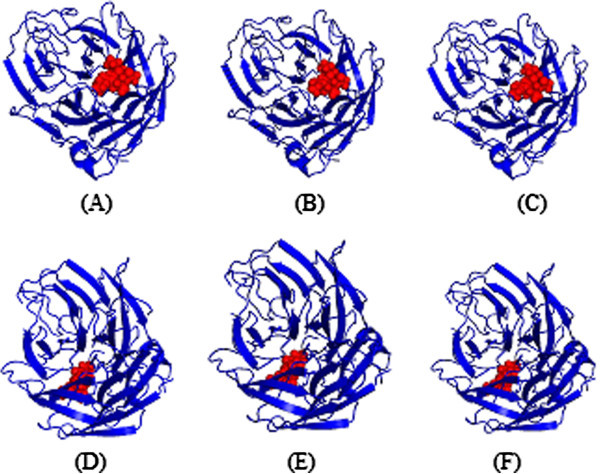
**Docked poses of oseltamivir with wild (A, B and C) and mutant type NA (D, E and F).**

**Figure 2:**
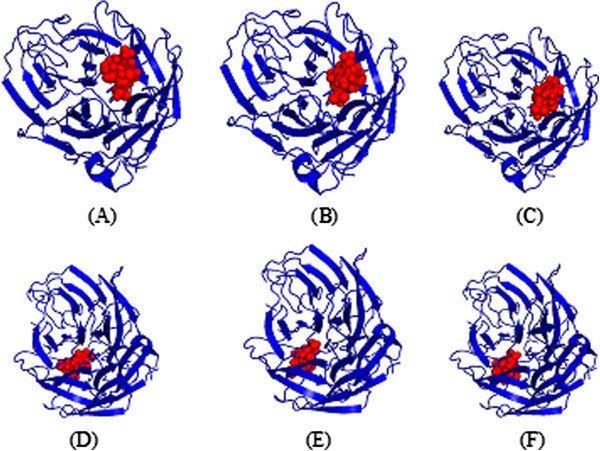
**Docked poses of neoglucobrassicin with wild (A, B and C) and mutant type NA (D, E and F).**

The docking study employed to identify the binding affinity of the molecule with the target proteins. However, standard error in the scoring function could leads to the improper selection of the lead compound. Thus, re-docking was employed to eliminate the false positive in the screening process. Then, X-ray crystallography structures of wild and mutant type NA were retrieved from the Protein Data Bank (PDB). The corresponding PDB ids are 2HU4 and 3CL0 for the wild and mutant type of NA respectively. The binding residues of the target structures were collected from the available literature evidences (Colman et al. 1983; Rungrotmongkol et al. 2009). Docking describes a process by which small molecule and the active site of biological macromolecule fit together in three-dimensional space and has contributed important proceedings to drug discovery for many years. Initially flexible docking simulation was performed using ArgusLab 4.0.1 (http://www.ArgusLab.com). We docked all the 4 molecules filtered from bioavailability analysis to the wild (2HU4) and mutant type of NA (3CL0) and the results were ranked by scoring function. The results of molecular docking showed that neoglucobrassicin, a compound obtained from *Brassica oleracea var. gongylodes*, was identified because it ranked first in the docking results (Table 2). Subsequently, re-docking was performed to eliminate the false positive compound from the results. The results were shown in Table 2 and the corresponding docked poses of NA-oseltamivir complexes and NA-neoglucobrassicin complexes of both wild and mutant type were shown in the Figures 1 and 2. It is interesting to observe that the average binding energy of NA-neoglucobrassicin is higher than NA-oseltamivir complex in both wild and mutant type of NA.

**Table 2:** **Comparison of free energy of binding between mutant type of NA with oseltamivir and virtual compounds obtained from traditional Chinese medicine database**

S.No	Ligand informations	Free energy of binding, ΔG (kcal/mol)							
		Wild type NA-Ligand complex				Mutant type NA-Ligand complex			
		Docking 1	Docking 2	Docking 3	Avg	Docking 1	Docking 2	Docking 3	Avg
1	Oseltamivir	-8.62	-8.43	-8.22	-8.42	-7.72	-7.54	-7.21	-7.49
2	Neogleucobrassicin	-10.48	-10.26	-10.37	-10.37	-8.5	-8.53	-8.72	-8.58
3	Acanthoglabrolide	-6.45	-6.38	-6.43	-6.42	-7.22	-7.19	-6.98	-7.13
4	AllylPropyldisulfide	-6.54	-6.76	-6.84	-6.713	-6.45	-6.434	-6.36	-6.414
5	Allyl monosulfide	-6.04	-6.25	-6.534	-6.27	-6.0	-6.0	-6.0	-6.14

**Figure 3:**
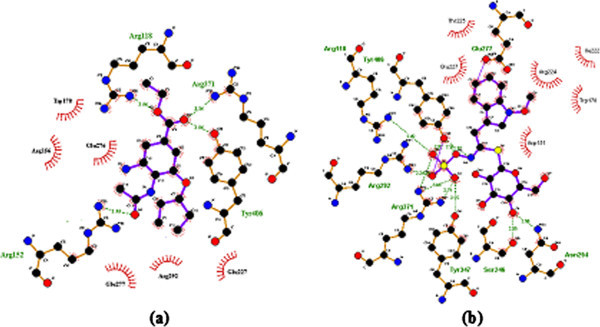
**The interaction of Oseltamivir (a) and Neoglucobrassicin (b) with wild type NA (PDB Code 2HU4).**

**Figure 4:**
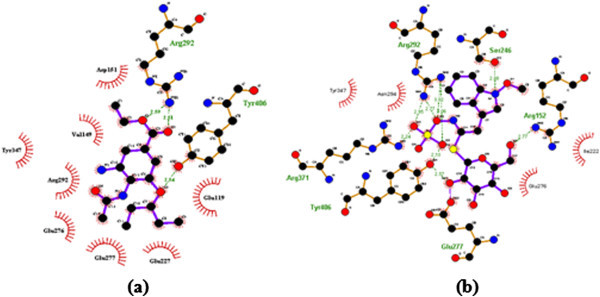
**The interaction of Oseltamivir (a) and Neoglucobrassicin (b) with mutant type NA (PDB Code 3CL0).**

LIGPLOT (Wallace et al. 1995) used to visualize the interactions exists in the complex structures. The docking conformation and the interacting pattern of oseltamivir and neoglucobrassicin with wild and mutant type NA were shown in Figures 3 and 4. It is evident from the figure that H274Y mutation alters the confirmations of the NA binding pocket. As a result the number of interactions between oseltamivir with mutant type NA was reduced and it leads to decrease in the binding affinity, thus confer drug resistance. Despite the H274Y mutation in the structure of NA, neoglucobrassicin were retained the interaction with the residues R-371 and R-152 as like the wild type NA. Furthermore, the existence of additional hydrogen bond in the residues R-292 and E-277 may also contribute to the effective binding of NA-neoglucobrassicin complex. Since there are several hydroxyl groups in neoglucobrassicin, they have the potential to form hydrogen bonds with surrounding residues (Figure 4). Hence, these extensive interactions are most important for the stability of NA-neoglucobrassicin complex.

### Molecular dynamics simulation

**Figure 5:**
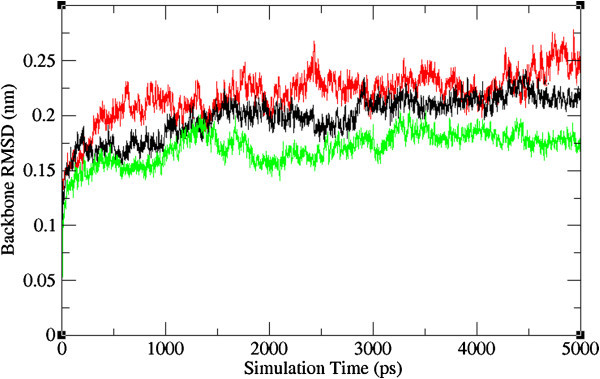
**Backbone root mean square deviations correspond to wild-oseltamivir (black), mutant-oseltamivir (red) and mutant- neoglucobrassicin (green) along the MD simulation at 300k.**

**Figure 6:**
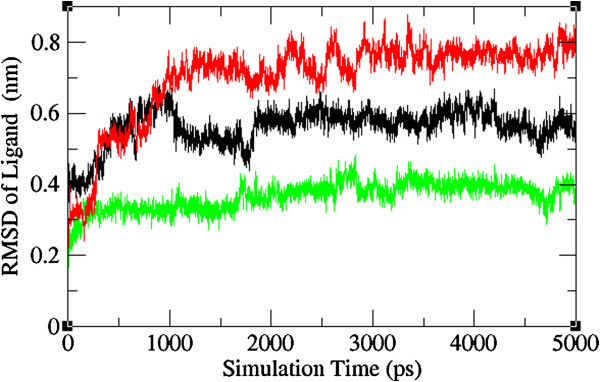
**Root mean square deviations correspond to ligand in NA-ligand complexes: wild-oseltamivir (black), mutant-oseltamivir (red) and mutant- neoglucobrassicin (green) along the MD simulation at 300k.**

The ligand binding and the important macromolecular motions associated with it are microscopic events that take place in mere millionths of a second. Therefore, a complete understanding of the atomistic energetics and mechanics of binding is unattainable using current experimental techniques. Molecular dynamics simulations are useful for filling in the details where experimental methods cannot (Durrant & McCammon 2011). Hence, in the present study molecular dynamic simulation was carried out by GROMACS package 4.5.3 (Hess et al. 2008; Spoel et al. 2005) adopting the GROMOS43a1 force field parameters which aimed to simulate the induced fit including potential conformational movements of both the protein and the ligand. Results showed that the average atom especially atoms of the NA-neoglucobrassicin complex movements were small, fast convergence of energy and changes in geometry as measured by Backbone RMSD analysis. Furthermore, we have analyzed the RMSD profile of the ligand in the protein-ligand complex structures to validate the stability of the lead compound. The RMSD results indicate that neoglucobrassicin movement is small in the mutant type of NA structure than Oseltamivir in the mutant type of NA. This is the indicative of neoglucobrassicin stability within the binding pocket of mutant type NA (Figures 5 and 6). This highlights the stable binding of the neoglucobrassicin with wild and mutant type NA.

### Toxicity and physicochemical properties

Many drug candidate fail in the clinical trials reasons is unrelated in the potency against the intended drug target. Pharmacokinetic and toxicity issues are blamed for more than half of all failure in the clinical trials. Therefore it is essential to evaluates Pharmacokinetic and toxicity of small molecules.

The parameter clogP and logS were assessed to analyze the pharmacokinetic property of the filtered set of compounds. clogP is a well-established measure of the compound’s hydrophilicity. Low hydrophilicities and therefore high log P values may cause poor absorption or permeation. It has been shown for compounds to have a reasonable probability of being well absorb their logP value must not be greater than 5.0. On this basis, neoglucobrassicin have log P values in the acceptable criteria.

Drug solubility is an important factor that affects the movement of a drug from the site of administration into the blood. It is known that insufficient solubility of drug can leads to poor absorption (Muegge 2003). Our estimated log S value is a unit stripped logarithm (base 10) of a compound’s solubility measured in mol/liter. There are more than 80% of the drugs on the market have an (estimated) log S value greater than -4. Table 3 shows solubility of oseltamivir and other virtual compounds. It is clear from the table that the solubility of neoglucobrassicin was found in the comparable zone with that of standard drugs to fulfill the requirements of solubility and could be considered as a candidate drug for oral absorption.

**Table 3:** **Toxicity risks and physicochemical properties of oseltamivir and virtual compounds predicted by OSIRIS property explorer**

S.No	Compound	Mutagenic	Tumorigenic	Irritant	cLogP	Solubility	Drug likeness	Drug score
1	Oseltamivir	No	No	No	1.439	-2.448	-1.499	0.535
2	Neoglucobrassicin	No	No	No	0.175	-4.006	-0.046	0.558
3	Epifriedelanol Acetate	No	No	No	7.729	-7.327	-12.317	0.134
4	g-strophanthin	No	No	No	2.138	-4.339	-0.327	0.422
5	Acanthoglabrolide	Medium	No	High	4.405	-4.187	-12.317	0.134
6	AllylPropyl disulfide	No	No	No	0.888	-2.767	-5.032	0.471
7	Allyl mono sulfide	No	No	No	0.714	-2.671	-3.852	0.481
8	Strictinin	No	No	No	0.89	-2.79	-2.836	0.284
9	Strophanthin	No	No	High	1.105	-3.79	-3.276	0.239
10	Acanthoside	No	No	No	1.787	-1.7	-1.884	0.259

### Drug likeness

The drug likeliness is another important parameter. Because drug like molecules exhibit favorable absorption, distribution, metabolism, excretion, toxicological (ADMET) parameters. Currently, there are many approaches to assess a compound drug-likeness based on topological descriptors, fingerprints of molecular drug-likeness structure keys or other properties such as clog P and molecular weight (Tetko 2005). In this work, Osiris program (Sander) was used for calculating the fragment-based drug-likeness of of oseltamivir and other virtual compounds. The drug-likeness value of neoglucobrassicin was found to be in acceptable criteria.

### Toxicity

The toxicity risk predictor locates fragments within a molecule, which indicate a potential toxicity risk. Toxicity risk alerts are an indication that the drawn structure may be harmful concerning the risk category specified. From the data evaluated in Table 3 indicates that the allyl propyldisulfide, allyl monosulfide and neoglucobrassicin non-mutagenic, non-irritating with no Tumorigenic effects when run through the mutagenicity assessment system comparable with standard drugs used.

### Drug score

**Figure 7:**
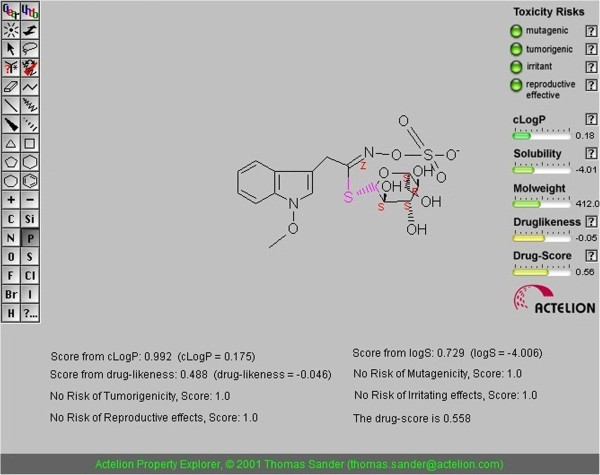
**Osiris property explorer showing drug likeness properties of Neoglucobrassicin.**

We have also examined the overall drug score (DS) for all the neoglucobrassicin and compared with that of standard drugs Oseltamivir. The drug score combines drug likeness, miLogP, log S, molecular weight and toxicity risks in one handy value than may be used to judge the compound’s overall potential to qualify for a drug. The result is shown in Table 3. Neoglucobrassicin showed good DS as compared with other lead molecules and standard drug used. The toxicity, drug-likeness and drug-score results for the neoglucobrassicin were illustrated in Figure 7.

## Conclusion

Neoglucobrassicin is a compound extracted from TCMD was predicted to be more potent inhibitor of NA than the existing drugs such as Oseltamivir. Favorable binding interactions with wild and mutant type of NA have been observed through an extensive docking study. Furthermore, RMSD analysis observed during MD simulation results certainly indicates the stable binding of neoglucobrassicin with NA structures. Moreover, neoglucobrassicin compound is based on natural product metabolites and are not expected to have undesirable side effects as the traditional drugs. This is also confirmed from our ADME and toxicity studies. We believed that the findings reported here might provide useful clues for designing powerful drugs against drug resistant target of NA.
